# Supplementary material for: Automated pipeline framework for processing of large-scale building energy time series data
Source: PLoS One. 2020 Dec 1;15(12):e0240461. doi: 10.1371/journal.pone.0240461 (PMC7707597; doi:10.1371/journal.pone.0240461)
Supplement: Attachment — Submitted filename: Plos One Reviewer Comments.pdf [file pone.0240461.s002.pdf]

Reviewer #1: The manuscript demonstrates a computing method that claims to be faster than others in order to sort and clean building energy data. The manuscript is generally well written and the topic of study is within the scope of the journal. It is suggested accepting this paper after minor revisions concerning the following comments and questions.

1. In the abstract, it is mentioned that this pipeline can analyze the data. In the body of the paper, there is no discussed methodology on how to analyze energy data. For example, HVAC has factors, such as the fan speed, coils, return air temperature, space temperature, discharge air temperature and others that need to be considered and weighted to analyze HVACs. It is suggested to just mention data cleaning and sorting at this stage.

We added a sentence to clarify that the pipeline can enable any data-driven analysis, and we have shown its performance with analyzing the HVAC scheduling.
"Our developed pipeline enables any applied analysis to be distributed to all the datasets and results stored in the database, automatically."

2. In the introduction, last paragraph, "data automatically goes through multiple processing", how data automatically goes through the process?

The use of "automatically goes through the process" is revised in the paper and now reads:
"In this paper, we demonstrate the development of an energy analytics pipeline wherein data, after being queried, automatically passes through multiple preprocessing, cleaning, assembly and ingestion steps in high performance and parallel computing environment with fast-track, smart and interactive capabilities."

3. It is suggested to explain if this pipeline can sort data live-online with connecting to some global controls, or like some other commissioning software, data need to be uploaded. Having high-speed computing capability along with live-online data analyzing tools are considerable value propositions.

The reviewer points out there is increased value sith capability of live-online data analyzing, which is very true, and our pipeline enables such a capability. We elaborated it as:
"Our developed pipeline addresses the automation, scalability and efficiency challenges of large-scale time-series processing, hence, can be utilized in live buildings data analysis applications."

4. The author should consider explaining his definition of automated analytics.

An explanation regarding automated analytics has been added,
"The automated process which implies the automatic flow of building data to each step of the pipeline, is designed to do all the required steps for data processing and analytics automatically

with an efficient framework and unified structure, and generate comparable results with data from different sources and formats."

> *5. Most figures have a long description. It is suggested to shorten the titles and add explanations to the text.*

According to the recommendation of the reviewer, we updated the explanations of some of the figures.

> *Reviewer #2: This paper proposes an automated pipeline building energy analytics -- BEA --a pipeline for virtual energy audit applied to time series. Authors use data sets in a non relational data warehouse in high-performance computing to manage job scheduling for a parallel processing. An interesting contribution is the protocol to assemble datasets and prepare for analysis. It also includes tools for data qualification that enhances data quality, and a machine learning algorithm: hierarchical clustering. The reported scalability is impressive.*
